# Supplementary material for: Multi‐dataset identification of innovative feature genes and molecular mechanisms in keratoconus
Source: J Cell Mol Med. 2024 Sep 19;28(18):e70079. doi: 10.1111/jcmm.70079 (PMC11412914; doi:10.1111/jcmm.70079)
Supplement: Supplementary file 2 — Material S2. [file JCMM-28-e70079-s002.zip › 7.GSEA analysis/5.RNF217-AS1/KEGG_O_GLYCAN_BIOSYNTHESIS.html]

Details for gene set KEGG\_O\_GLYCAN\_BIOSYNTHESIS[GSEA]

|  || Dataset | RNF217-AS1.group.cls#h\_versus\_l |
| Phenotype | group.cls#h\_versus\_l |
| Upregulated in class | l |
| GeneSet | KEGG\_O\_GLYCAN\_BIOSYNTHESIS |
| Enrichment Score (ES) | -0.5335015 |
| Normalized Enrichment Score (NES) | -1.4831955 |
| Nominal p-value | 0.060728744 |
| FDR q-value | 0.3222941 |
| FWER p-Value | 0.781 |
Table: GSEA Results Summary

  

Fig 1: Enrichment plot: KEGG\_O\_GLYCAN\_BIOSYNTHESIS      
 Profile of the Running ES Score & Positions of GeneSet Members on the Rank Ordered List

  

| SYMBOL | TITLE | RANK IN GENE LIST | RANK METRIC SCORE | RUNNING ES | CORE ENRICHMENT || 1 | GALNTL6 | na | 4283 | 0.242 | -0.0708 | No |
| 2 | B3GNT6 | na | 5455 | 0.201 | -0.0608 | No |
| 3 | GALNT6 | na | 8646 | 0.113 | -0.1281 | No |
| 4 | GALNT12 | na | 9300 | 0.099 | -0.1254 | No |
| 5 | GALNT5 | na | 10009 | 0.086 | -0.1270 | No |
| 6 | ST3GAL1 | na | 10984 | 0.070 | -0.1398 | No |
| 7 | GALNT2 | na | 11455 | 0.064 | -0.1395 | No |
| 8 | ST6GALNAC1 | na | 12479 | 0.049 | -0.1584 | No |
| 9 | GALNT3 | na | 12760 | 0.045 | -0.1566 | No |
| 10 | GALNT9 | na | 14154 | 0.029 | -0.1905 | No |
| 11 | C1GALT1 | na | 14351 | 0.026 | -0.1904 | No |
| 12 | GALNT8 | na | 16263 | 0.006 | -0.2441 | No |
| 13 | B4GALT5 | na | 16687 | 0.002 | -0.2559 | No |
| 14 | C1GALT1C1 | na | 18982 | -0.020 | -0.3177 | No |
| 15 | GALNT15 | na | 19311 | -0.023 | -0.3220 | No |
| 16 | GALNT10 | na | 19368 | -0.024 | -0.3184 | No |
| 17 | GALNT7 | na | 22133 | -0.052 | -0.3867 | No |
| 18 | GALNT1 | na | 25095 | -0.092 | -0.4521 | No |
| 19 | GALNTL5 | na | 25145 | -0.093 | -0.4333 | No |
| 20 | GALNT18 | na | 27103 | -0.128 | -0.4619 | No |
| 21 | GALNT11 | na | 27489 | -0.136 | -0.4433 | No |
| 22 | ST3GAL2 | na | 30619 | -0.228 | -0.4840 | Yes |
| 23 | GALNT4 | na | 31146 | -0.248 | -0.4453 | Yes |
| 24 | GCNT3 | na | 32841 | -0.349 | -0.4183 | Yes |
| 25 | GALNT13 | na | 33243 | -0.380 | -0.3472 | Yes |
| 26 | GALNT16 | na | 33603 | -0.420 | -0.2663 | Yes |
| 27 | GCNT4 | na | 33626 | -0.423 | -0.1749 | Yes |
| 28 | GALNT14 | na | 33748 | -0.440 | -0.0827 | Yes |
| 29 | GCNT1 | na | 34133 | -0.510 | 0.0171 | Yes |
Table: GSEA details [plain text format]

  

Fig 2: KEGG\_O\_GLYCAN\_BIOSYNTHESIS      
 Blue-Pink O' Gram in the Space of the Analyzed GeneSet

  

Fig 3: KEGG\_O\_GLYCAN\_BIOSYNTHESIS: Random ES distribution      
 Gene set null distribution of ES for **KEGG\_O\_GLYCAN\_BIOSYNTHESIS**
